# Coping strategies in anxious surgical patients

**DOI:** 10.1186/s12913-016-1492-5

**Published:** 2016-07-12

**Authors:** Hansjoerg Aust, Dirk Rüsch, Maike Schuster, Theresa Sturm, Felix Brehm, Yvonne Nestoriuc

**Affiliations:** Philipps-University of Marburg (UKGM StO Marburg), Marburg, Germany; Department of Psychosomatic Medicine and Psychotherapy, University Medical Center, Hamburg-Eppendorf, Martinistrasse 52, 20246 Hamburg, Germany; Department of Anesthesiology and Intensive Care; Philipps-University of Marburg, Baldingerstrasse, D-35033 Marburg, Germany

**Keywords:** Anxiety, Coping skills, Coping behaviour, Anaesthesia, Preoperative period, Patient care

## Abstract

**Background:**

Anaesthesia and surgery provoke preoperative anxiety and stress. Patients try to regain control of their emotions by using coping efforts. Coping may be more effective if supported by specific strategies or external utilities. This study is the first to analyse coping strategies in a large population of patients with high preoperative anxiety.

**Methods:**

We assessed preoperative anxiety and coping preferences in a consecutive sample of 3087 surgical patients using validated scales (Amsterdam Preoperative Anxiety and Information Scale/Visual Analogue Scale). In the subsample of patients with high preoperative anxiety, patients’ dispositional coping style was determined and patients’ coping efforts were studied by having patients rate their agreement with 9 different coping efforts on a four point Likert scale. Statistical analysis included correlational analysis between dispositional coping styles, coping efforts and other variables such as sociodemographic data. Statistical significance was considered for *p* < 0.05.

**Results:**

The final analysis included 1205 patients with high preoperative anxiety. According to the initial self-assessment, about two thirds of the patients believed that information would help them to cope with their anxiety (“monitors”); the remainder declined further education/information and reported self-distraction to be most helpful to cope with anxiety (“blunters”). There was no significant difference between these two groups in anxiety scores.

Educational conversation was the coping effort rated highest in monitors whereas calming conversation was the coping effort rated highest in blunters. Coping follows no demographic rules but is influenced by the level of education. Anxiolytic Medication showed no reliable correlation to monitoring and blunting disposition. Both groups showed an exactly identical agreement with this coping effort.

Demand for medical anxiolysis, blunting or the desire for more conversation may indicate increased anxiety. The use of the internet was independent of the anxiety level and the demand of information.

**Conclusion:**

Conversation with medical staff proved to be the most popular coping strategy. Acknowledgment of the division between information-seeking and blunting-like personalities is central to supporting the patient’s individual coping efforts. Internet access may be the easiest way to support coping today.

**Electronic supplementary material:**

The online version of this article (doi:10.1186/s12913-016-1492-5) contains supplementary material, which is available to authorized users.

## Background

Patients scheduled for anaesthesia and surgery often become anxious and stressed. They have to adapt or cope with this situation in order to regain control of their emotions [1]. Otherwise, such stress may exceed the individual resources of the patients and may negatively affect recovery from surgery [2–4]. Patients try to adopt or cope with this situation by using specific affective, cognitive and behavioural efforts to manage the demands of the actual situation. The stabilizing responses during stressful periods can be summarised within the concept of coping [1, 5, 6]. The individual coping style is generally related to the personality of the patient (dispositional coping style) while the used steps of action are mostly situationally adapted (coping efforts). Coping is therefore a function of personality and situational demands in order to regain emotional control [7, 8]. According to the stress model of Lazarus, a stressor itself does not primarily provoke stress. Rather, stress is a result of the subjective appraisal of the threat [9]. Coping may therefore be more effective if supported comprehensively by health care providers, especially in patients with high preoperative anxiety.

Much research into coping dates from the 1980s. Due to the rapid development of communication technology, many social aspects of life have since changed and so patterns of coping efforts may also have changed. In order to help patients cope effectively today, we must first understand their preoperative coping efforts and informational preferences.

We designed this study to obtain descriptive data about coping in the environment of a modern pre-anaesthetic clinic. Second, we wanted to analyse the role of internet and digital media on today’s coping behaviour. Due to the known wide spectrum of individual coping efforts in the literature and our aim to analyse data with impact on daily clinical life, we concentrated on patients with high preoperative anxiety and coping efforts which can be modified in a supportive way.

This is the first report of preferred coping efforts and their correlates in a large population of surgical patients with high preoperative anxiety.

## Methods

### Ethics approval

This study was approved by the local ethics committee (Ethics committee of the University Hospital of Marburg, Germany, Chairperson Prof. G. Richter; Correspondence dated February 2012; Approval No.: 18/12, dated March 1^st^ 2012). Due to its voluntary character, we obtained only verbal agreement to take part in the study-completion of the multipage questionnaire was equated with consent. Patients were advised, verbally and in print on the questionnaire cover page, that the completion of the questionnaire could be stopped at any time without giving a reason.

### Study subjects and data collection

All adult patients scheduled to undergo any kind of anaesthesia and surgeries were eligible to take part in this survey. Exclusion criteria included insufficient knowledge of the German language, impaired visual acuity and mental disorders which would prevent patients from being able to fill in a questionnaire. Questionnaires were completed at the pre-anaesthetic assessment clinic at Giessen Marburg University Hospital (UKGM)–Marburg campus while patients were waiting for the appointment with the physician. Two medical students who were participating in the study stood by to answer any questions concerning the questionnaire. We divided patients into those with low and those with high preoperative anxiety according to their anxiety score in the Amsterdam Preoperative Anxiety and Information Scale (see below: *anxiety scoring*).

### Study design

#### Sociodemographic and clinical variables

Patients’ age, gender, education, previous experience of surgery and anaesthesia, cancer surgery and any subsequent physical impairment were assessed.

#### Anxiety scoring

We used two validated tools to evaluate preoperative anxiety, namely the German version [10] of the Amsterdam Preoperative Anxiety and Information Scale (APAIS) and the visual analogue scale (VAS) ranging from 0 (not at all) to 10 (extreme) [11–13]. Due to the fact that the APAIS consists of two sub scales referring to anxiety and information (both concerning anaesthesia and surgery separately), we calculated and discussed our results concerning APAIS-total (APAIS-T), APAIS-anxiety subscale (APAIS-A) and APAIS-information subscale (APAIS-I). The English version of the APAIS is shown in the Additional file 1. By analogy, the VAS had to be completed twice (one regarding fear of anaesthesia (VAS-A) and one for fear of surgery (VAS-S) in order to be able to discriminate between these two components of preoperative anxiety). Patients who had a APAIS-A score > 10 were classified as patients with high anxiety according to Moerman et al. [11].

#### Coping assessment

Patients dispositional coping style was ascertained with the two items of “seeking-” or “preventing information” in accordance with monitoring or blunting coping-styles introduced by Miller in 1983 [14].

Situational coping efforts were assessed by 9 questions, specifically designed for this study in cooperation with the Department of Clinical Psychology and Psychotherapy, University of Marburg [Table 1]. Patients were asked to rate their preferences regarding these 9 coping efforts on a four point Likert scale: disagreement, slight agreement, mainly in agreement and full agreement. The coping efforts were categorised into three dimensions: problem-focused, emotion-focused and social-support seeking according to Bruchon-Schweitzer et al. [15].

**Table 1:** Coping efforts and coping dimensions

Problem- focused coping	I would like to be able to use the internet to gain further information. *(Internet)* ^a^
	I would like to have educational software or video material on a PC for further information. *(Multimedia)* ^a^
	I would like to be offered alternative methods of stress reduction (e.g. Acupuncture). *(Alternative Medicine)* ^b^
Emotional-focused coping	I would like to receive anxiolytic drugs. *(Anxiolytic Medication)* ^c^
	I use mainly mental resources to cope. (’not thinking about it…“, I distract myself, “positive thinking …”). *(Mental Strategies)* ^a^
social support	I get the information I want from family members and friends. *(Family/Friends)* ^a^
	I research the reputation of the clinic and consult other patients treated there. *(Reputation)* ^a^
	I would like a serious and reassuring conversation with a doctor or nurse. *(Calming Conversation)* ^a^
	I would like to talk in some depth with a physician regarding the procedure. (*Physician (educational)*) ^a^

Note: The left column contains the coping dimensions and the right column lists the wording of the coping efforts used in the questionnaire. Allocation of the coping efforts are indicated by letters in superscript: a: Bruchon-Schweizer et al. (1996) [15]; b: Lavery et al. (1996) [26]; c: Perodeau et al. (2007) [29]

The main reasons for having chosen these coping efforts are listed below:

- *Internet:* The Internet provides a source of both further information and distraction, answers to questions which patients might hesitate to ask, and exemplifies medical issues. Given smartphone use is rapidly growing in all social classes [16, 17] no costly hardware is necessary apart from a public internet access-point.
- *Multimedia:* Edited educational films or interactive software offer more specific ways of providing further information on diagnosis, treatment, risk and safety aspects of medical procedures. Clinics are able to canalize the information according to their specific organizational and service portfolios. The only costs are those related to the production or purchase of educational films, specialized software and the appropriate hardware (computers, tablets, etc.).
- *Physician, educational:* Anaesthesia and surgery include the loss of privacy and patients must have confidence in their medical service providers. Given physicians play a key role in the treatment of patients, individual contact with a physician, preferably the treating physician, has the potential to be a strong confidence-building measure. In fact, a recent survey among patients concerning their preferences with respect to the pre-anaesthesia consultation demonstrated that personal contact with the attending physician is the patient’s greatest need [18].
- *Reputation:* Preoperative anxiety may be based to some extent on the uncertainty concerning the quality of the medical care of a hospital in general or the quality of a specific treatment (e.g. an operation) [19]. Accordingly, any positive statements from patients that have had any kind of treatment in the same hospital can have a calming effect. Understandably, positive reports from patients who have undergone the same procedure could have an even more calming effect.
- *Family/Friends:* Common sense suggests that people consult family members and friends (i.e. people they know and they can trust) rather than any person whose reliability they can’t assess [20, 21].
- *Calming Conversation:* Previous work suggests that a conversation does not have to convey very detailed information concerning a problem in order to minimise stress and anxiety. In fact the mere conversation itself may have a calming effect [22, 23].
- *Mental Efforts:* Mental coping revolves round the many ways in which a patient may regard the situation. Some patients prefer to be maximally informed about all aspects of a potentially threatening medical situation (e.g. surgery) and gather information. Others prefer to ignore information regarding the potentially threatening medical situation and gain strength by positive thinking or by rationalisation. About a third of all persons typically react with a blunting-like coping style. These so called blunters prefer to be distracted [24, 25].
- *Alternative Medicine:* Progressive muscle relaxation, homeopathy autogenic training or acupuncture may ease stress and restore emotional stability [26–28]).
- *Anxiolytic Medication:* The classic treatment of iatrogenic stress is the prescription of anxiolytic drugs. Such medications, mostly benzodiazepines, ease stress, cause retrograde amnesia and facilitate sleep [29, 30].

### Sample size

A study of preoperative anxiety (study 1) was designed and carried out in combination with this study of coping efforts concerning preoperative anxiety (study 2). The intention was to include at least 1000 patients with high anxiety (i.e. patients with an APAIS-A > 10 as defined by Moerman et al. [11]) into this study (study 2). A pilot study carried out at our hospital showed that about one third of patients have high anxiety (i.e. APAIS-A >10). Accordingly, 3000 patients needed to be enrolled in study 1 in order to get 1000 patients that qualify to be included in this study (study 2). To compensate for a possibly lower eligibility rate and drop outs we planned to enrol 3200 patients in study 1.

#### Data processing and statistics

In order to better differentiate the chosen coping efforts we used the Likert scale (disagreement = 1; slight agreement = 2, mainly agreement = 3 and full agreement = 4) for assessment. Data analysis was performed using SPSS® 20.0. (IBM Corporation). Data is presented in a descriptive manner, in absolute and percentage values as well as in means with standard deviation (SD), median and interquartile range (IQR). The psychometric properties of the APAIS and its subscales are given as correlations. To estimate the reliability of the APAIS and its subscales we calculated Cronbach’s alpha.

All correlational analyses were performed using alpha = 0.05. A hierarchical multiple regression analysis was performed to determine predictors of preoperative anxiety and fear of anaesthesia as measured by APAIS-A and both VAS (VAS-A and VAS-S). Prior to the regression analysis we checked for any multicollinearity effects using the variance inflation factor (VIF). No collinearities could be seen. The first step of the regression analysis included the basic data such as gender, age, education, number of previous surgical operations, cancer surgery, subsequent material physical impairment and prior experiences with anaesthesia. Coping-related variables as monitoring-like or blunting-like personality and all of the nine coping efforts were entered in the second step and the increased percentage of explained variances was calculated using delta *R*^*2*^*.*

## Results

We enrolled 3200 patients (study 1) between March 2012 and April 2013. Of these, 113 were excluded because of violation of inclusion criteria (4), contradictory answers (7), and incompleteness of APAIS testing (102) leaving 3087 patients for analysis. Of those, 1251 subjects (40.5%) had high anxiety (APAIS-A score > 10) who therefore were eligible for this study (study 2). In 46 out of these 1251 cases the coping section of the questionnaire was incomplete. The final analysis therefore included 1205 datasets. The mean APAIS-A of these patients with high anxiety was 13.5 ± 2.2 as against 7.55 ± 2.1 for the patients with low anxiety (Additional file 2). Characteristics of the patients with high anxiety are shown in Table 2.

**Table 2:** Sociodemographic and clinical variables of patients with high anxiety

Age (years)	49 ± 17
Female	860 (71)
Male	345 (29)
Education: ^a^	
≤9 years	419 (35)
10 years	432 (36)
≥13years	354 (29)
Previous surgeries:	
None	154 (13)
1–2	470 (39)
≥3	581 (48)
Malignant tumor^b^	173 (14)
Burdening physical impairment^c^	165 (14)
Prior experiences with anaesthesia:	
only good experiences	597 (49)
only bad experiences	85 (7)
both experiences	346 (29)
no experiences	177 (15)

Note. Data are presented as mean ± SD or as number and relative incidence (n, %) of group total. ^a^ Education includes education in school, college and university. ^b^ = Patients who are scheduled to undergo surgery of a malignant tumor. ^c^ Patients who are scheduled to undergo surgery involving a burdening impairment

### Psychometric properties of the APAIS, its two subscales and both VAS

APAIS-T correlated very highly with APAIS-A (*r* = 0.924) and highly with APAIS-I (*r* = 0.690). APAIS-T also correlated highly with both VAS (VAS-A: *r* = 0.729; VAS-S: *r* = 0.760). The three APAIS scales showed a high reliability (Cronbach’s Alpha: APAIS-T α = 0.830 APAIS-A α = 0.872 and APAIS-I α = 0.742).

### Coping style: information-seeking (monitoring-like) or information-avoiding (blunting-like)

According to the initial self-assessment, 63.7% of the patients believed that information would help them to cope with their anxiety; the remainder declined further education/information and reported self-distraction to be most helpful to cope with anxiety. These attitudes showed a strong negative correlation (Pearsons *r* = -0.856). There was no significant difference between these two groups in anxiety scores: (APAIS-T: 19.4 ± 3.0 vs. 20.1 ± 3.0APAIS-A: 13.45 ± 2.2 vs. 13.5 ± 2.2, VAS-A: 5.6 ± 2.4 vs. 5.6 ± 2.3, and VAS-S: 6.6 ± 2.1 vs. 6.5 ± 2.2.; for all *p* > 0.5) They did not differ concerning the following variables either: age, gender, number of previous surgeries, cancer surgery or surgery with subsequent physical impairment. However, those declining further information had significantly lower values in APAIS-I (*p* < 0.001), were less educated (*p* = 0.0002) and had fewer negative experiences with anaesthesia (*p* = 0.0106).

### Coping efforts

**Fig. 1:**
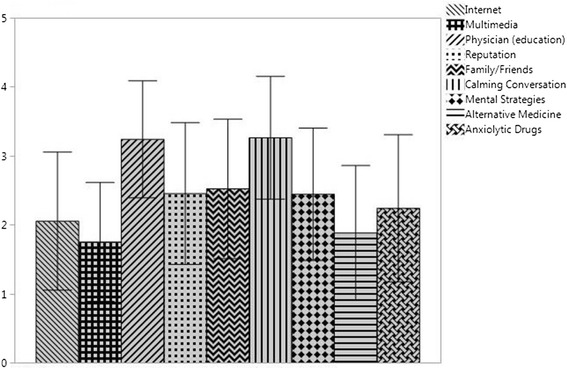
Frequency of chosen coping efforts. Note: Agreement displayed as Mean ± SD based on the results on a Likert-Scale

The magnitude of agreement with the different coping efforts is presented in Table 3 and Fig. 1.

**Table 3:** Agreement with coping efforts

	All patients (*n* = 1205)			Patients with monitoring-like coping style (*n* = 768)	Patients with blunting-like coping style (*n* = 437)
	Mean ± SD	Median	IQR	Mean ± SD	Mean ± SD
Internet	2.06 ± 1.00	2	2	2.24 ± 1.04	1.74 ± 0.86
Multimedia	1.76 ± 0.86	2	1	1.88 ± 0.88	1.54 ± 0.78
Physician (educational)	3.25 ± 0.85	3	1	3.49 ± 0.68	2.81 ± 0.94
Reputation	2.46 ± 1.03	3	1	2.58 ± 0.99	2.24 ± 1.05
Family/Friends	2.53 ± 1.01	3	1	2.60 ± 0.99	2.41 ± 1.00
Calming Conversation	3.27 ± 0.89	4	1	3.39 ± 0.82	3.05 ± 0.10
Mental Strategies	2.45 ± 1.00	3	1	2.30 ± 0.95	2.70 ± 0.90
Alternative Medicine	1.89 ± 0.98	2	1	1.98 ± 1.00	1.73 ± 0.91
Anxiolytic Medication	2.24 ± 1.07	2	2	2.24 ± 1.05	2.24 ± 1.13

Note: Agreement with coping efforts (Likert-Scale) 1 = disagreement; 2 = slight-, 3 = main- and 4 = full agreement – are displayed as mean ± SD; median and interquartile range (IQR)

Patients reported agreement with more than one coping effort. Correlations between coping efforts are shown in Additional file 3. Strongest correlations were seen between *Internet* and *Multimedia* (*r* = 0.66), *Reputation* and *Family/Friends* (*r* = 0.53), *Physician (educational)* and *Calming Conversation* (*r* = 0.45), *Physician (educational)* and *Reputation* (*r* = 0.33) and *Reputation* and *Calming Conversation* (*r* = 0.33). Both conversational coping efforts (educational and calming) were negatively correlated to *Mental Strategies* of coping.

### Correlations between coping efforts, sociodemographic data and monitoring coping style

Strongest correlations between coping efforts and sociodemographic variables were found for *Internet* and education (*rho* = 0.298) as well as *Internet* and age (*r* = -0.296). Results concerning all correlations are shown in Additional file 4. Significant (*p* < 0.001) correlations existed between education and *Internet* (*r* = 0.298) and Age and *Internet* source (*r* = -0.296).

### Coping efforts in the context of anxiety

Beside the coping effort *Internet* (*p* = 0.208) all other coping efforts were considered significantly more helpful by patients with high preoperative anxiety (*p* < 0.001) compared to patients with low anxiety. Therefore, the use of the internet proved to be independent of the anxiety level and the demand of information.

In order to better understand the chosen coping efforts in the context of high anxiety, it is important to differentiate between the anxiety and information parts of the APAIS. The APAIS-T correlated strongest to *Physician* (educational) (*r* = 0.297), *Calming conversation* (*r* = 0.254) and *Anxiolytic medication* (*r* = 0.228). Correlations differ for both APAIS subscales. APAIS-I had a stronger correlation to *Physician (educational)* (*r* = 0.427) and a weaker correlation with *Anxiolytic medication* (*r* = 0.033). APAIS-A had a stronger correlation with *Anxiolytic medication* (*r* = 0.293) and a weaker one to *Physician (educational)* (*r* = 0.088) (see Additional file 5).

A hierarchical multiple regression analysis was performed to predict the extent of preoperative anxiety and fear of anaesthesia as measured by APAIS-A. In order to prevent effects of multicollinearity the Variance Inflation Factor (VIF) was calculated. This proved to be for all predictors in a range from 1.053 to 4.808. Therefore multicollinearity could be ruled out.

In the first step of the regression analysis age, gender, education, previous experience of surgery and anaesthesia, cancer surgery and any subsequent physical impairment were entered into the calculation. We could show that 12.4% of the determined variance in APAIS-A could be explained by those patient characteristics (ΔR^2^ = 0.124, F (10.3008) = 42,443, *p* <0.001.). This value increased significantly in the prediction model by inclusion of the self-assessment (monitoring-like or blunting-like) and the nine coping efforts. The explained variance increased to 30.2%, ΔR^2^ = 0.179, F (11.2997) = 69.78 *p* <0.001. In particular, blunting-like personality β = 0.129, t (2997) = 5.138, *p* < .001, *Calming conversation* β = 0.125, t (2997) = 6.557, *p* <0.001 and *Anxiolytic Medication* β = 0.319, t (2997) = 19,393, *p* <0.001, contributed significantly to the prediction of surgical and anaesthesia anxiety (Additional file 6).

### Coping efforts - monitoring-like and blunting-like disposition

Patients with a monitoring-like personality showed a positive correlation to problem-focused and social-support coping. Informationally accented coping efforts such as *Physician (educational)* (*r* = 0.397) or *Internet* (*r* = 0.236) showed particularly intermediate correlations where as *Anxiolytic Medication* showed no correlation (*r* = -0.009) (see Additional file 4).

Blunting-like patients appeared to prefer emotional coping efforts such as *Mental Strategies* (*r* = 0.224) but were also not correlated to Anxiolytic Medication (*r* = 0.005).

## Discussion

The coping efforts considered most helpful among patients with high preoperative anxiety were efforts consisting of conversation with medical staff. Personal contact to the physician proved to be the most wanted source for information. This is remarkable given the development of the modern multimedia environment and the resulting changes in our social behaviour over recent decades. Except for *Internet*, all other coping efforts were considered to be more helpful in patients with high anxiety. This confirmed our primary intention to focus our interest regarding coping on patients with higher anxiety.

When analysing the 1205 patients with high anxiety (APAIS > 10) we first focused on patients’ personality. Miller and Mangan (1983) introduced the terms “monitors”/“monitoring” (information-seeking) and “blunters”/“blunting” (information-avoiding or distracting) to describe two basic dispositional coping approaches to threatening medical situations [14]. Recognition of these two basic approaches (i.e. attention toward the threat vs. cognitive avoidance) is thought to be essential for analysis of the individual response to threatening events [24, 25, 31]. A third of our patients with high preoperative anxiety proved to be patients with a blunting-like disposition who choose to avoid receiving detailed information. Interestingly, they did not differ in their mean anxiety level from those with monitoring-like personality, contradicting the results of other studies that have reported greater anxiety in monitors [14, 32]. However, since these studies did not focus on patients with high preoperative anxiety but included patients of all anxiety levels our results provide further insight into a large sample of specifically burdened patients. According to results of our multiple regression analysis, blunting-like, but not monitoring-like behaviour proved to be an independent predictor for greater anxiety according to results of multiple regression analysis. We assume that our study cohort – including more than one thousand patients with high preoperative anxiety in a real preoperative setting – provides valid data regarding the dysfunctional effect of blunting-strategies.

The ratio of 1 : 2 between blunting and monitoring personality that was described by Miller et al. 35 years ago [33, 34] was replicated within this study. Nevertheless, blunting and its consequences are still barely acknowledged in our pre-anaesthetic or pre surgical assessment clinics, possibly because little is known about active coping behaviours in monitors and blunters. Miller et al. [35] postulated that monitors do not use the information they seek for active behavioural coping and may display a negative correlation between monitoring and problem-focused coping, although this has been questioned by Bar-Tal et al. [36]. The latter assert that people who are predominantly blunters may be inclined to use more emotional regulation strategies. Monitors however prefer to cope with problems because this strategy includes the element of information-seeking in order to face a threat [36]. In that study, monitors correlated positively with problem-focused coping and social support-seeking, as also reported by Timmermans et al. in 2007 among patients with needing radiotherapy [37]. We are also able to confirm this with our data. In self-stated information-seeking (monitoring-like) patients of our study problem-focused and social support-coping efforts were all positively correlated (e.g. *Physician (educational) r* = 0.397). Information-avoiding (blunting-like) patients prefer emotional coping efforts like *Mental Strategies*. Accordingly monitoring-like behaviour and both emotional focused coping efforts had a negative correlation (e.g. *Mental Strategies r* = -0.224). Similar results have been reported by Rood et al. among patients with haematological malignancies [38]. They described a greater desire for more information and more involvement in decision-making in high-monitoring patients. In addition, their study showed no correlation between lower education and blunting strategies. Nevertheless Menaghan [39] and Billings and Moos [40] also reported that better education leads to more active and problem-focused coping.

While problem-focused coping aims to retain control e.g. through collecting more information, active coping in the form of seeking social support looks for assistance [7, 41, 42]. Despite the widespread use of multimedia, *Internet* is ranked only seventh and interactive *Multimedia* ninth. In contrast, the most appreciated coping efforts in our study proved to be conversational. This is important, because information presented without further individual reprocessing may increase stress rather than ease it [14]. While monitoring-like patients rated educational conversation with the physician highest (*Physician (educational)*) blunting-like patients considered *Calming Conversation* more helpful then educational conversation *(Physician (educational)*). Despite the continuing pressure to economise on staffing costs, the majority of patients still want individual conversation and attention to ease pre-operative stress. This echoes the findings of our previous study that individual contact with a physician, preferably the treating physician, is the most import pre-anaesthetic requirement for almost two thirds of patients [18]. Egbert et al. found that physician-patient conversation may be as calming as anxiolytic drugs [43]. In general, *Anxiolytic medication* is considered to be a coping effort belonging to emotion-focused coping, which is aimed to manage the associated emotions, rather than changing the threatening situation itself. Accordingly it could be expected that blunters compared to monitors assign *Anxiolytic Medication* higher ratings. Surprisingly, both groups showed an exactly identical agreement with this coping effort.

While there are no differences between monitoring-like and blunting-like behaviour concerning the demand for anxiolytic drugs, such differences do exist in the context of anxiety. The APAIS-Scale consists of two parts – one that assesses the patient’s anxiety and one that explores the patient’s need for information. Both results are combined to form the final score (APAIS-T) but it is important to assess both parts separately in order to interpret the score correctly. For instance, the analysis of the APAIS-T suggests that patients demand conversation (including educational or calming) and medical anxiolysis in the same manner. However, when focusing on patients with high scores in the information part only, anxiolytic medication becomes negligible and the demand for educational dialogue with a physician shows the highest correlation of all. In contrast, high APAIS-A has the strongest correlation with the desire for anxiolytic medication and communication to address the need for calming. This emphasis is also seen with high VAS-A and VAS-S.

### Coping support in pre-anaesthetic or pre-surgical assessment clinics

Anxiety may adversely affect anaesthetic care [44, 45], result in requiring larger doses of anaesthetics with more side effects [3, 46, 47], entail negative effects during recovery from surgery [48, 49], and cause postoperative physical complaints (e.g. headache, dizziness, nausea) [50]. We all develop strategies to cope with stress; a supportive environment may optimise the individual positive coping efforts, but such support has to be more than merely providing information. Studies of coping mechanisms need to take into account the difference in arousal when monitors and blunters are exposed to both their preferred and non-preferred efforts [14], because information without individual adaptive comment may be counter-productive [1].

Our main message is that, despite economic constraints, patient–staff conversation is still of paramount importance. Accordingly, medical staff should be trained in professional conversation techniques [51]. This survey has also demonstrated that it is crucial to discriminate the needs of blunting-like and monitoring-like patients]. Therefore, it is very important that physicians are aware of coping efforts in order to understand their patients’ needs and act accordingly, i.e., do not force information on patients who wish to receive as little information as possible but provide more information to those who seek it. In this context, physicians should be sensitive to verbal and nonverbal communication from patients regarding their coping method. It is also possible to question the patient first about the amount of information they request. Additionally, rationalising, positive thinking or distracting can be accepted, even supported, as long as the patient is initially made aware of the diagnosis and treatment. The waiting area of assessment clinics should be designed to allow both monitors and blunters to feel comfortable; information should be offered only on demand. Internet access may be acceptable to both groups; cost-intensive multimedia terminals, however, seem to be of no significance in this context.

Anxiolytic medication is a traditional method for easing preoperative anxiety or stress. Negative aspects include potential side effects such as drowsiness, loss of personal control and the risk of injuries, prolonged anaesthesia or inadequate affects due to wrong timing or dosage. Our results show that most patients do not expect anxiolytics to be the first line of defence. Other supported coping efforts should come first, with anxiolytics being held back for patients with greater anxiety. In case patients are considered to need anxiolytics than they should be administered in a sufficient dosage. Generally, anxiolytic medication should be routinely available, but at the patient’s request.

### Limitations

Due to the voluntary participation in this study we did not record the number of patients approached and the number of patients excluded (e.g. due to a language barrier). This may cause a bias of the study results if, for instance, patients with high anxiety were more likely to refuse to participate in the survey. Secondly, in the context of the wide range of possible individual coping efforts we decided to focus our approach on prevalidated specific coping efforts and adapted them to the hospital setting. We developed our own questionnaire based on the validated three-factor description of coping efforts by Bruchon-Schweizer et al. [15] (problem-focused, emotion focused and social-support seeking) in order to embrace as much as possible of the full range of supportable coping efforts most likely to be used by the patients. In other words, we do not claim completeness concerning the list of coping efforts.

Thirdly, although we have concentrated on the preoperative period in the environment of a pre-anaesthetic assessment clinic, coping is in fact a multidisciplinary issue throughout the perioperative period. We focused on coping behaviour to gain insights into future possible coping support, which will be investigated in additional studies.

We had to limit the number of questions asked. We addressed patients’ dispositional coping style of monitoring and blunting personality with two basic questions only and did not use “The Miller Behavioral Style Scale (MBSS)” [32]. Therefore all our results are only discussed in the context of a monitoring-like or blunting-like behaviour/personality.

## Conclusion

A third of our patients experience high anxiety and mental stress prior to anaesthesia and surgery. They use many coping efforts and hospitals should be able to support them with pharmacological and non-pharmacological strategies. Two thirds of patients with increased anxiety seek information related to surgery and anaesthesia while one third avoids such information. For all, the preferred coping strategy is a conversation with medical staff. The internet is used as information source by all patients independent of anxiety level. Refusal to receive information should be accepted as a legitimate aspect of coping. Coping follows no demographic rules but is influenced by the level of education. Patients who request anxiolytic medication, who refuse further information or show an increased need for conversation may be suffering from severe anxiety and their needs should be taken seriously.

## Abbreviations

APAIS, Amsterdam preoperative anxiety and information scale; APAIS-A, APAIS-anxiety sub scales; APAIS-I, APAIS-information sub scales; e.g., exempli gratia; IQR, interquartile range; m, median; n, number, amount; PC, personal computer; SD, standard deviation; VAS, visual analogue scale; VIF, variance inflation factor; Y.I., constant (Y Intercept)

## Additional files

Additional file 1: The Amsterdam Preoperative Anxiety and Information Scale (APAIS). This supplement shows the APAIS questionnaire. (DOCX 31 kb) Additional file 2: APAIS and VAS scores. This supplement shows a table presenting mean anxiety levels (APAIS and VAS scores) of all patients (*n* = 3087) and the subset of patients with high anxiety (*n* = 1205). (DOCX 27 kb) Additional file 3: Correlations between coping efforts. This supplement shows a table with correlations between the coping efforts. Correlation coefficients (Pearson/Spearman) and the corresponding significances are presented. (DOCX 29 kb) Additional file 4: Correlations between coping efforts, sociodemographic data and monitoring coping style. This supplement shows a table with correlations between coping efforts, sociodemographic data and monitoring-like personality. Correlation coefficients and the corresponding significances are displayed. (DOCX 28 kb) Additional file 5: Correlations between coping efforts and anxiety-scales (APAIS and VAS). This supplement shows a table with correlations between coping efforts and APAIS- and VAS-scores. Correlation coefficients and the corresponding significances are presented. (DOCX 29 kb) Additional file 6: Multiple hierarchical regression analysis. This supplement shows a table summarizing results of the multiple hierarchical regression analysis concerning the contribution of various patient characteristics to the prediction of surgical and anaesthesia anxiety. (DOCX 39 kb)
