# Supplementary material for: Coping strategies in anxious surgical patients
Source: BMC Health Serv Res. 2016 Jul 12;16:250. doi: 10.1186/s12913-016-1492-5 (PMC4941033; doi:10.1186/s12913-016-1492-5)
Supplement: Additional file 1: — The Amsterdam Preoperative Anxiety and Information Scale (APAIS). This supplement shows the APAIS questionnaire. (DOCX 31 kb) [file 12913_2016_1492_MOESM1_ESM.docx]

Additional file 1:

**The** **Amsterdam Preoperative Anxiety and Information Scale (APAIS)**

|  | Not at all | 1 | 2 | 3 | 4 | 5 | Extremely |
| --- | --- | --- | --- | --- | --- | --- | --- |
|  | | | | | | | |
| 1. I am worried about the anesthetic |  | 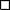 | 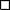 | 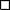 | 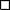 | 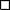 |  |
| 2. The anesthetic is on my mind  continually |  | 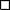 | 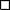 | 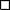 | 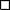 | 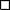 |  |
| 3. I would like to know as much as  possible about the anesthetic |  | 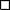 | 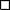 | 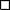 | 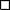 | 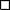 |  |
| 4. I am worried about the procedure |  | 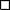 | 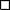 | 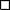 | 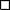 | 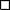 |  |
| 5. The procedure is on my mind  continually |  | 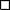 | 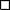 | 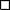 | 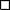 | 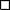 |  |
| 6. I would like to know as much as  possible about the procedure |  | 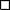 | 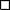 | 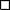 | 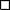 | 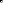 |  |

The subscales:

Anesthesia-related anxiety = 1 + 2

Surgery-related anxiety = 4 + 5

Information desire component = 3 + 6 (= APAIS-I)

Anxiety component = 1 + 2 + 4 + 5 (= APAIS-A)
