# Supplementary material for: Coping strategies in anxious surgical patients
Source: BMC Health Serv Res. 2016 Jul 12;16:250. doi: 10.1186/s12913-016-1492-5 (PMC4941033; doi:10.1186/s12913-016-1492-5)
Supplement: Additional file 2: — APAIS and VAS scores. This supplement shows a table presenting mean anxiety levels (APAIS and VAS scores) of all patients (n = 3087) and the subset of patients with high anxiety (n = 1205). (DOCX 27 kb) [file 12913_2016_1492_MOESM2_ESM.docx]

Additional file 2: APAIS and VAS scores

|  | **APAIS-A** | **APAIS-I** | **APAIS-T** | **VAS-A** | **VAS-S** |
| --- | --- | --- | --- | --- | --- |
| All patients  (n=3087) | 9.9 ± 3.6 | 6.3 ± 1.8 | 16 ± 4.6 | 3.5 ± 2.7 | 4.3 ± 2.8 |
| Patients with high anxiety  (n=1205) | 13.5 ± 2.2 | 6.9 ± 1.8 | 20 ± 3.1 | 5.6 ± 2.4 | 6.6 ± 2.2 |

Note: Data are presented as mean ± SD.
