# Supplementary material for: Coping strategies in anxious surgical patients
Source: BMC Health Serv Res. 2016 Jul 12;16:250. doi: 10.1186/s12913-016-1492-5 (PMC4941033; doi:10.1186/s12913-016-1492-5)
Supplement: Additional file 3: — Correlations between coping efforts. This supplement shows a table with correlations between the coping efforts. Correlation coefficients (Pearson/Spearman) and the corresponding significances are presented. (DOCX 29 kb) [file 12913_2016_1492_MOESM3_ESM.docx]

Additional file 3: Correlations between coping efforts

|  |  | **Multimedia** | **Physician (edu-cational)** | **Repu-tation** | **Family/ Friends** | **Calming Conver-sation** | **Mental Strategies** | **Alter--native Medicine** | **Anxio-lytic Drugs** |
| --- | --- | --- | --- | --- | --- | --- | --- | --- | --- |
| Internet |  | 0.658  (<0.001) | 0.279  (<0.001) | 0.295  (<0.001) | 0.256  (<0.001) | 0.180  (<0.001) | -0.075  (0.010) | 0.242  (<0.001) | 0.037  (0.196) |
| Multimedia |  | 1 | 0.237  (<0.001) | 0.289  (<0.001) | 0.206  (<0.001) | 0.125  (<0.001) | -0.023  (0.429) | 0.256  (<0.001) | 0.063  (0.030) |
| Physician (educational) |  |  | 1 | 0.325  (<0.001) | 0.212  (<0.001) | 0.447  (<0.001) | -0.134  (<0.001) | 0.190  (<0.001) | 0.054  (0.059) |
| Reputation |  | | | 1 | 0.528  (<0.001) | 0.329  (<0.001) | -0.016  (0.572) | 0.252  (<0.001) | 0.106  (<0.001) |
| Family/ Friends |  | | | | 1 | 0.264  (<0.001) | -0.046  (0.112) | 0.205  (<0.001) | 0.090  (0.002) |
| Calming Conversation |  | | | | | 1 | -0.130  (<0.001) | 0.198  (<0.001) | (0.180  (<0.001) |
| Mental Strategies |  | | | | | | 1 | 0.003  (0.925) | 0.003  (0.917) |
| Alternative Medicine |  | | | | | | | 1 | 0.161  (<0.001) |

Note: Data represent correlation coefficients (Pearson / Spearman) and the corresponding significances in parentheses
