# Supplementary material for: Coping strategies in anxious surgical patients
Source: BMC Health Serv Res. 2016 Jul 12;16:250. doi: 10.1186/s12913-016-1492-5 (PMC4941033; doi:10.1186/s12913-016-1492-5)
Supplement: Additional file 4: — Correlations between coping efforts, sociodemographic data and monitoring coping style. This supplement shows a table with correlations between coping efforts, sociodemographic data and monitoring-like personality. Correlation coefficients and the corresponding significances are displayed. (DOCX 28 kb) [file 12913_2016_1492_MOESM4_ESM.docx]

Additional file 4: Correlations between coping efforts, sociodemographic data and monitoring coping style

|  | **Monitoring-like**  **Personality** | **Age** | **gender** | **Education** |
| --- | --- | --- | --- | --- |
| Internet | 0.236  (<0.001) | - 0.296  (<0.001) | - 0.029  (0.321) | 0.298  (<0.001) |
| Multimedia | 0.189  (<0.001) | - 0.183  (<0.001) | - 0.094  (0.001) | 0.181  (<0.001) |
| Physician (educational) | 0.397  (<0.001) | - 0.041  (0.156) | 0.025  (0.382) | 0.129  (<0.001) |
| Reputation | 0.155  (<0.001) | -0.033  (0.257) | 0.024  (0.411) | 0.102  (<0.001) |
| Family/Friends | 0.075  (0.012) | - 0.148  (<0.001) | 0.016  (0.579) | 0.092  (0.001) |
| Calming Conversation | 0.181  (<0.001 | - 0.034  (0.235) | 0.119  (0,000) | 0.086  (0.003) |
| Mental Strategies | - 0.224  (<0.001) | 0.083  (0.004) | 0.009  (0.749) | - 0.076  (0.008) |
| Alternative Medicine | 0.140  (<0.001) | - 0.038  (0.183) | 0.021  (0.459) | 0.131  (<0.001) |
| Anxiolytic Medication | - 0.009  (0.756) | 0.019  (0.508) | 0.060  (0.037) | - 0.043  (0.135) |

Note: For statistical analysis information seeking was set as 1 and avoidance as 0; age was analysed in context of older age; gender was set with male=0 and female=1; education was analysed in context of higher education. We displayed correlations and the corresponding Significances (p): Pearson (Significance) / Spearman-Rho (Significance)
