# Supplementary material for: Coping strategies in anxious surgical patients
Source: BMC Health Serv Res. 2016 Jul 12;16:250. doi: 10.1186/s12913-016-1492-5 (PMC4941033; doi:10.1186/s12913-016-1492-5)
Supplement: Additional file 5: — Correlations between coping efforts and anxiety-scales (APAIS and VAS). This supplement shows a table with correlations between coping efforts and APAIS- and VAS-scores. Correlation coefficients and the corresponding significances are presented. (DOCX 29 kb) [file 12913_2016_1492_MOESM5_ESM.docx]

Additional file 5: Correlations between coping efforts and anxiety-scales (APAIS / VAS)

|  | **APAIS-**  **anxiety** | **APAIS-**  **information** | **APAIS**  **total** | **VAS-anxiety-**  **anaesthesiology** | **VAS-anxiety-**  **surgery** |
| --- | --- | --- | --- | --- | --- |
| Internet Source | 0.015  (0.608) | 0.268  (<0.001) | 0.163  (<0.001) | 0.038  (0.188) | 0.032  (0.271) |
| Multimedia | 0.016  (0.590) | 0.245  (<0.001) | 0.153  (<0.001) | 0.034  (0.236) | 0.021  (0.457) |
| Physician (educational) | 0.088  0.002 | 0.427  (<0.001) | 0.297  (<0.001) | 0.106  (<0.001) | 0.076  (0.008) |
| Reputation | 0.100  (<0.001) | 0.236  (<0.001) | 0.200  (<0.001) | 0.073  (0.011) | 0.118  (<0.001) |
| Family / Friends | 0.069  0.016 | 0.169  (<0.001) | 0.149  (<0.001) | 0.080  (0.005) | 0.091  (0.001) |
| Calming Conversation | 0.163  (<0.001) | 0.242  (<0.001) | 0.254  (<0.001) | 0.167  (<0.001) | 0.172  (<0.001) |
| Mental Strategies | - 0.034  (0.244) | - 0.105  (<0.001) | - 0.088  (0.02) | <0.001  (0.995) | -0.071  0.013 |
| Alternative Medicine | 0.083  (0.004) | 0.110  (<0.001) | 0.119  (<0.001) | 0.095  (0.001) | 0.066  (0.022) |
| Anxiolytic Medication | 0.293  (<0.001) | 0.033  (0.246) | 0.228  (<0.001) | 0.283  (<0.001) | 0.244  (<0.001) |

Note: We correlated the coping efforts to raising scores as APAIS/VAS. Higher correlation therefore describe coping in context of higher anxiety. We displayed correlations (Pearson) and the corresponding Significances (p): Pearson (Significance)
