# Supplementary material for: Coping strategies in anxious surgical patients
Source: BMC Health Serv Res. 2016 Jul 12;16:250. doi: 10.1186/s12913-016-1492-5 (PMC4941033; doi:10.1186/s12913-016-1492-5)
Supplement: Additional file 6: — Multiple hierarchical regression analysis. This supplement shows a table summarizing results of the multiple hierarchical regression analysis concerning the contribution of various patient characteristics to the prediction of surgical and anaesthesia anxiety. (DOCX 39 kb) [file 12913_2016_1492_MOESM6_ESM.docx]

Additional file 6: Multiple hierarchical regression analysis

| Modell | | unstandardized coefficient | | standardized coefficient | T | Signi-ficance | 95%  confidence interval (B) | | collinearity | |
| --- | --- | --- | --- | --- | --- | --- | --- | --- | --- | --- |
|  | | B | standard deviation | beta |  |  | lower confidence bound | upper confidence bound | Tolerance | VIF |
| First step | Y.I. | 10.259 | 0.310 |  | 33.121 | <0.001 | 9.652 | 10.866 |  |  |
|  | gender | 1.895 | 0.127 | 0.259 | 14.865 | <0.001 | 1.645 | 2.145 | 0.957 | 1.044 |
|  | age | -0.012 | 0.004 | -0.059 | -3.027 | 0.002 | -0.021 | -0.004 | 0.769 | 1.301 |
|  | edu. (1) | -0.296 | 0.156 | -0.039 | -1.894 | 0.058 | -0.603 | 0.010 | 0.690 | 1.450 |
|  | edu. (2) | -0.384 | 0.166 | -0.049 | -2.319 | 0.020 | -0.709 | -0.059 | 0.647 | 1.546 |
|  | PreSg. (1) | -0.520 | 0.217 | -0.070 | -2.399 | 0.016 | -0.944 | -0.095 | 0.346 | 2.889 |
|  | PreSg. (2) | -0.992 | 0.217 | -0.137 | -4.567 | <0.001 | -1.419 | -0.566 | 0.323 | 3.100 |
|  | malign. | 0.533 | 0.196 | 0.050 | 2.716 | 0.007 | 0.148 | 0.918 | 0.860 | 1.162 |
|  | impair. | 0.872 | 0.209 | 0.075 | 4.175 | <0.001 | 0.462 | 1.281 | 0.897 | 1.115 |
|  | PrevGood. | -0.547 | 0.159 | -0.061 | -3.442 | 0.001 | -0.858 | -0.235 | 0.941 | 1.063 |
|  | PrevBad. | 1.299 | 0.142 | 0.160 | 9.160 | <0.001 | 1.021 | 1.577 | 0.955 | 1.047 |
|  | | | | | | | | | | |
| Sec  ond step | Y.I. | 5.880 | 0.399 |  | 14.751 | <0.001 | 5.098 | 6.661 |  |  |
|  | gender | 1.305 | 0.117 | 0.179 | 11.147 | <0.001 | 1.075 | 1.534 | 0.907 | 1.102 |
|  | age | -0.008 | 0.004 | -0.038 | -2.134 | 0.033 | -0.016 | -0.001 | 0.723 | 1.383 |
|  | edu. (1) | -0.274 | 0.141 | -0.036 | -1.945 | 0.052 | -0.551 | 0.002 | 0.677 | 1.476 |
|  | edu. (2) | -0.314 | 0.154 | -0.040 | -2.033 | 0.042 | -0.617 | -0.011 | 0.596 | 1.679 |
|  | PreSg. (1) | -0.488 | 0.194 | -0.065 | -2.515 | 0.012 | -0.868 | -0.107 | 0.345 | 2.902 |
|  | PreSg. (2) | -0.905 | 0.195 | -0.125 | -4.635 | <0.001 | -1.288 | -0.522 | 0.319 | 3.132 |
|  | malign. | 0.291 | 0.176 | 0.027 | 1.653 | 0.098 | -0.054 | 0.637 | 0.854 | 1.171 |
|  | impair. | 0.519 | 0.187 | 0.045 | 2.771 | 0.006 | 0.152 | 0.886 | 0.890 | 1.124 |
|  | PrevGood. | -0.546 | 0.142 | -0.061 | -3.834 | <0.001 | -0.826 | -0.267 | 0.934 | 1.070 |
|  | PrevBad. | 0.810 | 0.129 | 0.100 | 6.275 | <0.001 | 0.557 | 1.063 | 0.921 | 1.086 |
|  | monit. | 0.544 | 0.193 | 0.073 | 2.816 | 0.005 | 0.165 | 0.923 | 0.344 | 2.905 |
|  | blunt. | 0.998 | 0.194 | 0.129 | 5.138 | <0.001 | 0.617 | 1.379 | 0.369 | 2.707 |
|  | www | -0.193 | 0.075 | -0.056 | -2.590 | 0.010 | -0.339 | -0.047 | 0.498 | 2.009 |
|  | F/P | 0.087 | 0.086 | 0.021 | 1.016 | 0.309 | -0.081 | 0.256 | 0.564 | 1.773 |
|  | PhsyEd. | 0.243 | 0.074 | 0.064 | 3.279 | 0.001 | 0.098 | 0.388 | 0.620 | 1.613 |
|  | Reput. | 0.053 | 0.067 | 0.015 | .783 | 0.434 | -0.079 | 0.185 | 0.603 | 1.658 |
|  | FamFrds. | 0.180 | 0.066 | 0.052 | 2.729 | 0.006 | 0.051 | 0.310 | 0.630 | 1.586 |
|  | C.C. | 0.429 | 0.065 | 0.125 | 6.557 | <0.001 | 0.301 | 0.557 | 0.641 | 1.561 |
|  | M.S. | -.244 | 0.055 | -0.069 | -4.447 | <0.001 | 0-.352 | -0.137 | 0.956 | 1.046 |
|  | AltMed. | 0.087 | 0.066 | 0.022 | 1.320 | 0.187 | 0-.042 | 0.216 | 0.821 | 1.218 |
|  | AnxMed. | 1.159 | 0.060 | 0.319 | 19.393 | <0.001 | 1.042 | 1.276 | 0.862 | 1.161 |

Note: Y.I.: Constant (Y Intercept); age: age (years); edu. (1): education: ≤9 vs. 10 years; edu. (2): education: ≤9 vs. ≥13years; PreSg. (1): number of previous surgeries: None vs. 1–2; PreSg. (2) number of previous surgeries: None vs 1–2 vs ≥3; malign.: surgery of malignant tumor; impair.: subsequent burdening physical impairment; PrevGood.: good experiences with anaesthesia; PrevBad.: bad experiences with anaesthesia; monit.: Information-seeking (monitoring-like); blunt.: Information-preventing (blunting-like); www: Internet ; F/P: Film/PC (Multimedia); PhsyEd.: Physician (educational); Reput.: Reputation; FamFrds.: Family / Friends; C.C.: Calming Conversation; M.S.: Mental Strategies; AltMed.: Alternative Medicine; AnxMed.: Anxiolytic Medication; VIF: Variance Inflation Factor
